# Antibody-dependent infection of human macrophages by severe acute respiratory syndrome coronavirus

**DOI:** 10.1186/1743-422X-11-82

**Published:** 2014-05-06

**Authors:** Ming Shum Yip, Nancy Hiu Lan Leung, Chung Yan Cheung, Ping Hung Li, Horace Hok Yeung Lee, Marc Daëron, Joseph Sriyal Malik Peiris, Roberto Bruzzone, Martial Jaume

**Affiliations:** 1 HKU-Pasteur Research Pole and Center of Influenza Research, School of Public Health, LKS Faculty of Medicine, The University of Hong Kong, Hong Kong, Hong Kong SAR; 2 Department of Microbiology, LKS Faculty of Medicine, The University of Hong Kong, Hong Kong, Hong Kong SAR; 3 Departement of Immunology, Institut Pasteur, Paris, France; 4 Centre d’Immunologie de Marseille-Luminy, Inserm U1104, Marseille, France; 5 Department of Cell Biology and Infection, Institut Pasteur, Paris, France

**Keywords:** SARS-CoV, Spike, Antibody-dependent enhancement, Macrophage, Fcγ receptor, Antibodies, Pseudotypes

## Abstract

**Background:**

Public health risks associated to infection by human coronaviruses remain considerable and vaccination is a key option for preventing the resurgence of severe acute respiratory syndrome coronavirus (SARS-CoV). We have previously reported that antibodies elicited by a SARS-CoV vaccine candidate based on recombinant, full-length SARS-CoV Spike-protein trimers, trigger infection of immune cell lines. These observations prompted us to investigate the molecular mechanisms and responses to antibody-mediated infection in human macrophages.

**Methods:**

We have used primary human immune cells to evaluate their susceptibility to infection by SARS-CoV in the presence of anti-Spike antibodies. Fluorescence microscopy and real-time quantitative reverse transcriptase polymerase chain reaction (RT-PCR) were utilized to assess occurrence and consequences of infection. To gain insight into the underlying molecular mechanism, we performed mutational analysis with a series of truncated and chimeric constructs of fragment crystallizable γ receptors (FcγR), which bind antibody-coated pathogens.

**Results:**

We show here that anti-Spike immune serum increased infection of human monocyte-derived macrophages by replication-competent SARS-CoV as well as Spike-pseudotyped lentiviral particles (SARS-CoVpp). Macrophages infected with SARS-CoV, however, did not support productive replication of the virus. Purified anti-viral IgGs, but not other soluble factor(s) from heat-inactivated mouse immune serum, were sufficient to enhance infection. Antibody-mediated infection was dependent on signaling-competent members of the human FcγRII family, which were shown to confer susceptibility to otherwise naïve ST486 cells, as binding of immune complexes to cell surface FcγRII was necessary but not sufficient to trigger antibody-dependent enhancement (ADE) of infection. Furthermore, only FcγRII with intact cytoplasmic signaling domains were competent to sustain ADE of SARS-CoVpp infection, thus providing additional information on the role of downstream signaling by FcγRII.

**Conclusions:**

These results demonstrate that human macrophages can be infected by SARS-CoV as a result of IgG-mediated ADE and indicate that this infection route requires signaling pathways activated downstream of binding to FcγRII receptors.

## Background

The continuous threat of respiratory viruses to public health was exemplified by the global impact of the SARS-CoV outbreak in 2003 [1], by the occurrence since 2003 of confirmed human cases of H5N1 avian influenza in many countries, particularly across Asia [2], and the 2009 H1N1 influenza pandemic [3]. The recent emergence in the Arab peninsula of a novel coronavirus responsible for the Middle East respiratory syndrome (MERS-CoV), [4,5] and the new H7N9 strain of avian influenza that has jumped into humans [6,7] in China underscore the need to continue work in this direction.

It is now agreed that SARS-CoV can infect not only the respiratory tract, but can also affect other organ systems and several reports have demonstrated infection of hematopoietic cells [8-10]; however, the mechanism by which SARS-CoV enters into immune cells, which do not express the SARS-CoV receptor angiotensin-converting enzyme 2 (ACE2) [11,12] has remained poorly understood. Both C-type lectin receptors such as liver/lymph node-specific intercellular adhesion molecule-3-grabbing integrin (L-SIGN) or dendritic cell specific intercellular adhesion molecule 3-grabbing non-integrin (DC-SIGN) [13,14], as well as antibody-mediated infection may provide SARS-CoV with an opportunity to modify its tropism.

Because of the lack of effective antiviral strategies to control coronaviruses infections, vaccination is still regarded as a major option for preventing resurgence of SARS and related diseases. We previously showed that a SARS-CoV vaccine candidate based on recombinant, full-length SARS-CoV Spike-protein trimers triggered infection of human B cell lines despite eliciting in vivo a neutralizing and protective immune response in rodents [15]. More recently, we demonstrated that anti-Spike antibody potentiates infection of both monocytic and lymphoid immune cell lines, not only by SARS-CoVpp but also by replication-competent SARS-coronavirus [16], thus providing evidence for a novel and versatile mechanism by which SARS-CoV can enter into target cells that do not express the conventional ACE2 virus receptor and are otherwise refractory to the virus. Such infection pathway may have implications for understanding the tropism and pathogenesis of the virus and, therefore, we further investigated the molecular and cellular mechanisms underlying ADE of SARS-CoV infection.

By monitoring the susceptibility of human bulk primary immune cells (i.e. peripheral blood mononuclear cells) we have established the occurrence of ADE of SARS-CoVpp infection in different circulating immune cell types, among which the monocytic lineage (CD68^+^ cells) was the primary target. In addition to monocytes, human macrophages were also infected by SARS-CoV in presence of anti-Spike antibodies only. ADE-mediated infection of macrophages, however, did not support productive replication of the virus. Finally, we have provided evidence that the intracellular signaling motif – but not the IgG binding motif – of the FcγR is the key molecular determinant for triggering ADE of SARS-CoVpp. Our findings conclusively demonstrate that anti-spike serum promotes internalization of SARS-CoV by human macrophages.

## Results

### Primary human macrophages are susceptible to SARS-CoV infection through antibody-mediated pathway

**Figure 1:**
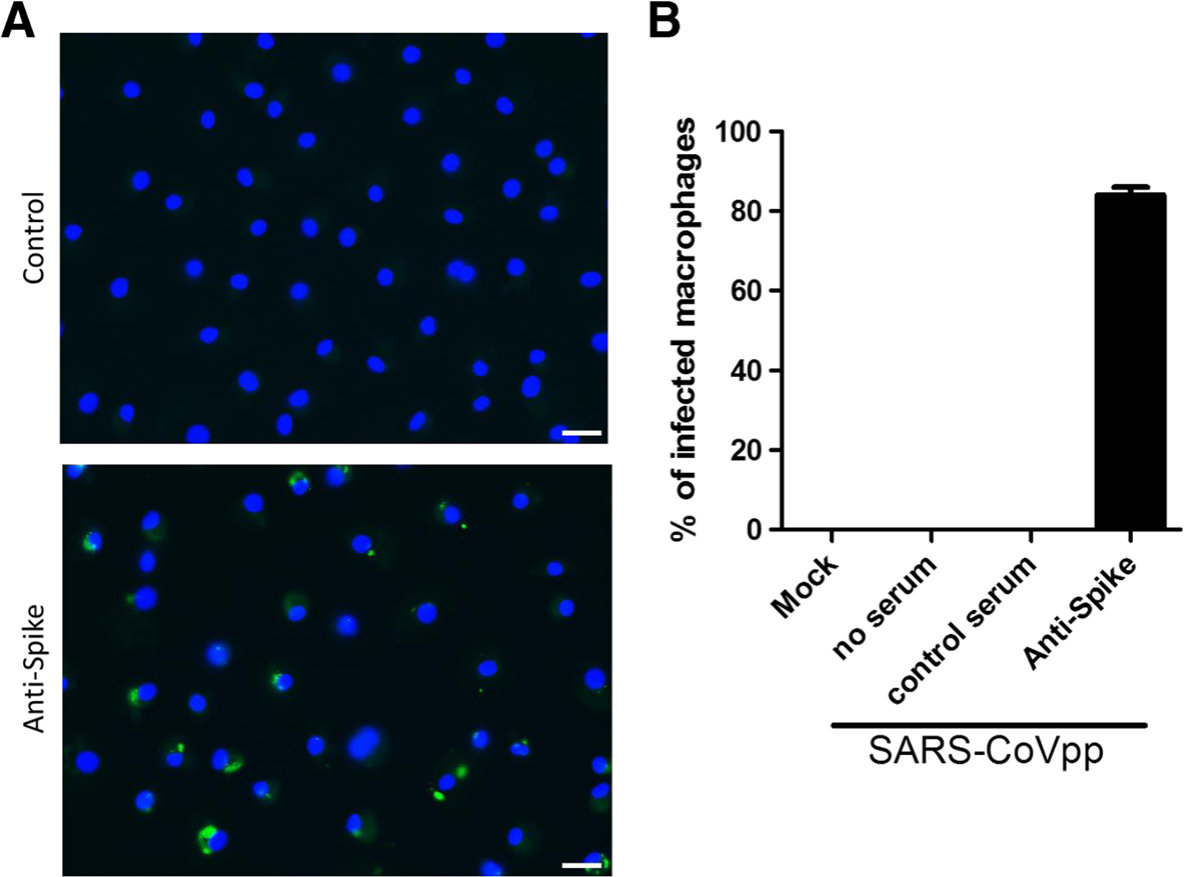
**Anti-Spike but not control serum triggered infection of human macrophages by SARS-CoVpp. (A)** Monocyte-derived macrophages were incubated for 1 hour with SARS-CoVpp carrying a luciferase reporter gene in the presence of a 1:1000 dilution of either anti-Spike or control serum and infected cells were detected by fluorescence staining of firefly luciferase (green), as described under Materials and methods. Cell nuclei were labeled with DAPI (blue). SARS-CoVpp infection of macrophages occurred only in the presence of anti-Spike serum. Images are representatives of five independent experiments using macrophages from five donors. **(B)** Infection was quantified by counting the percentage of immunofluorescence-positive cells in randomly chosen fields using Metamorph software. Results are shown as means ± SEM. Scale bar: 20 μm.

**Figure 2:**
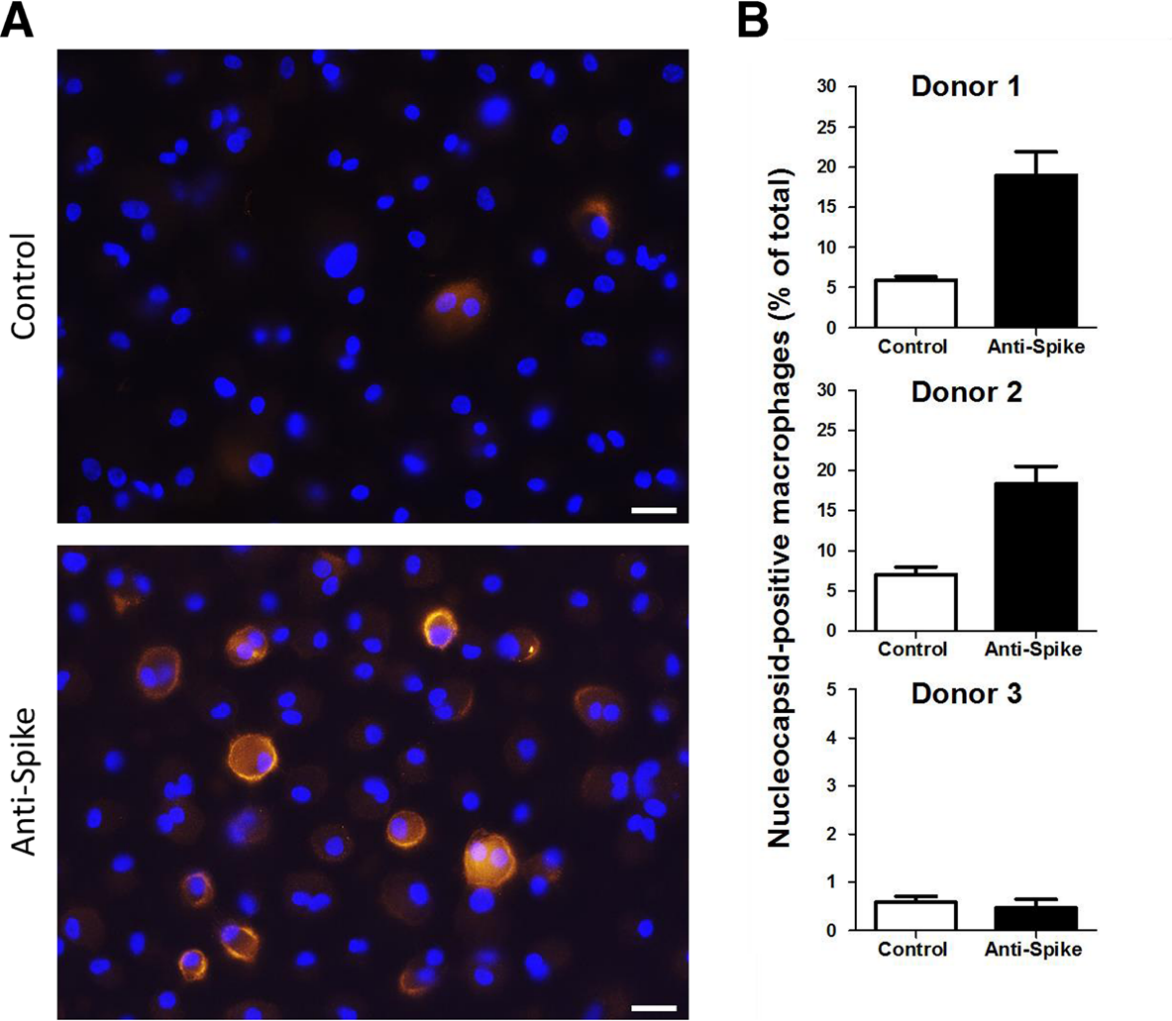
**Anti-Spike serum enhanced SARS-CoV infection in human monocyte-derived macrophages. (A)** Monocyte-derived macrophages were infected with SARS-CoV (strain HK39849) at an MOI of 1 in the presence of a 1:1000 dilution of either anti-Spike (black bars) or control (white bars) serum for 1 hour and then cultured with fresh medium for 6 hours as described under Materials and methods. After fixation, cells were permeabilized for intracellular staining of SARS-CoV nucleocapsid protein, which was revealed by TRITC-conjugated goat anti-mouse antibody (orange) while cell nuclei were stained with DAPI (blue). Images shown are representatives of the results obtained with macrophages from three donors at 6 hours post infection. Scale bar: 20 μm. **(B)** Infectivity of SARS-CoV was determined by calculating the percentage of nucleocapsid-positive cells in five randomly selected fields using Metamorph software. Presence of anti-Spike serum increased infectivity of SARS-CoV by 3-4-fold in macrophages of two out of three donors. Note that the ordinate’s scale for Donor 3 is different for ease of visualization. Results are shown as means ± SEM.

**Figure 3:**
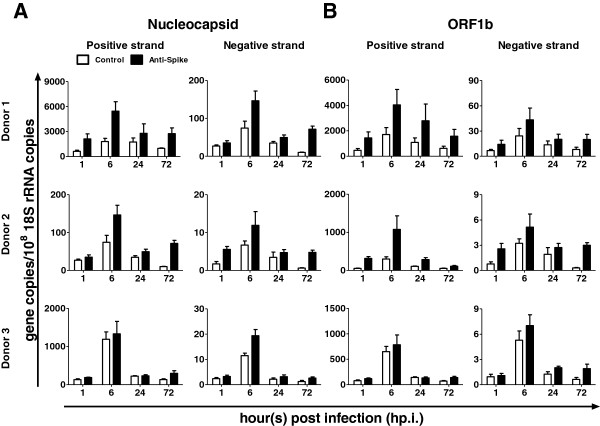
**Nucleocapsid and ORF1b gene expression in SARS-CoV infected human macrophages.** Monocyte-derived macrophages from three independent donors were infected with SARS-CoV as described in the legend to Figure 2 in the presence of anti-Spike (black bars) or control serum (white bars). Positive and negative RNA strands were determined for SARS-CoV nucleocapsid **(A)** and ORF1b **(B)** at the specified time points (hours) post infection (h.p.i.) using real-time qPCR. Data are shown as the number of viral RNA copies normalized to that of 10^8^ copies of 18S rRNA in the corresponding sample. Overall, RNA levels were higher in infected cells in presence of anti-Spike compared to control serum. Results are shown as means ± SEM of four measurements from two separate runs for each donor and gene.

Because our previous observations revealed that a human monocytic cell line THP-1 was susceptible to ADE of infection [16], we investigated the occurrence of ADE of infection in primary human macrophages in vitro, firstly by taking advantage of SARS-CoVpp that can be safely used to mimic the viral entry process [15,17]. We found that SARS-CoVpp opsonized with a 1:1000 dilution of anti-Spike serum readily infected over 80% of primary human macrophages, as determined by immunofluorescence staining of firefly luciferase at 72 hours post infection (h.p.i.). By contrast, cells exposed to SARS-CoVpp opsonized with control serum did not show any positive staining of the luciferase reporter protein (Figure 1). These experiments extend our previous observations and indicate that anti-Spike antibodies facilitate infection of SARS-CoVpp into human macrophages. We next tested whether this altered tropism was also displayed by replication-competent SARS-CoV. Human macrophages were infected at a multiplicity of infection (MOI) of 1 with the same dilutions of anti-Spike or control immune serum and the infection pattern was examined by both immunofluorescence staining of SARS-CoV nucleocapsid protein and real-time quantitative PCR measurement of viral RNAs. Positive immunofluorescence signals were detected only at 6 h.p.i. when SARS-CoV was opsonized with both control and anti-Spike serum (Figure 2). Interestingly, whereas presence of control serum led to only modest infection by SARS-CoV (~5% of cells), a 4-fold increase in the percentage of positive cells was noted in the presence of anti-Spike serum for two out of three donors (Figure 2). Such enhanced infection pattern was paralleled by the number of viral gene copies measured (Figure 3). Thus, compared to inoculums containing control serum, there was a 2 to 3-fold increase in the detection of both positive and negative strands of viral genes in macrophages infected in presence of anti-Spike serum, which was more pronounced at 1 and 6 h.p.i. There was a rapid decline in viral RNA from 6 to 24 h.p.i. for both conditions and no further changes were detected at later time points (Figure 3). However, it should be noted that the primer set used for the nucleocapsid gene could also detect the negative strand of the viral genomic RNA and could not distinguish it from sub-genomic material. In addition, we measured by real-time quantitative PCR copies of both viral nucleocapsid and ORF1b genes in culture supernatants to determine the release of SARS-CoV particles from the infected macrophages. Copy numbers of both viral RNAs, however, remained unchanged at all the selected time points during the course of the experiment (less than 200 copies/μl), regardless of whether macrophages had been infected in the presence of either control or anti-Spike serum (data not shown).

### ADE of SARS-CoVpp infection is dependent on the dose of IgG fraction of heat-inactivated immune-serum

**Figure 4:**
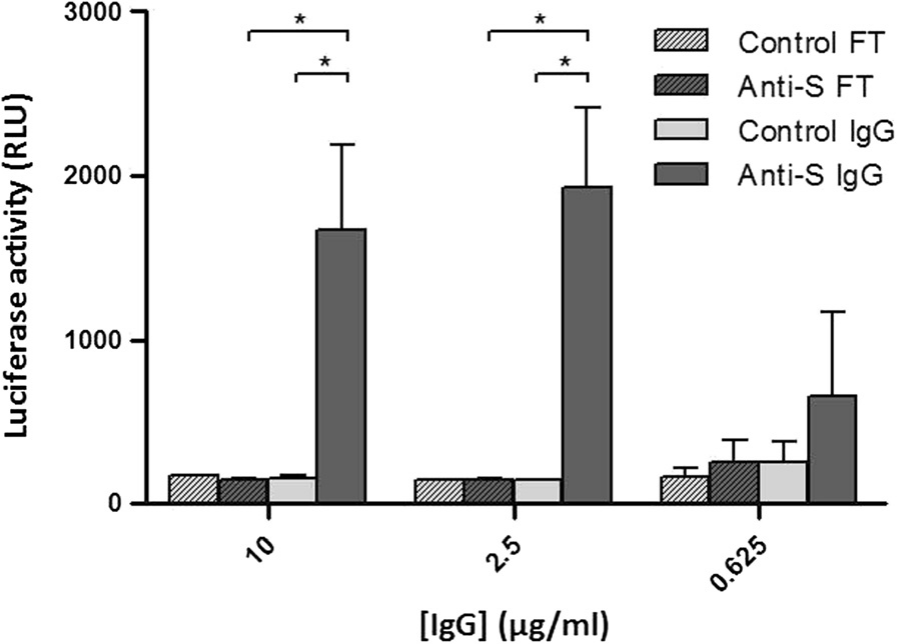
**Purified IgGs from heat-inactivated mouse anti-Spike serum mediate ADE of infection of Raji cells by SARS-CoVpp.** Serial dilutions of purified IgGs (solid bars) and protein G-sepharose column flow-through (hatched bars) from either heat-inactivated mouse anti-Spike (dark grey) or control (light grey) serum were incubated with SARS-CoVpp for 1 hour and opsonized particles were then used to infect Raji cells as described under Materials and methods. SARS-CoVpp infection was assessed three days post infection by expressing luciferase activity as Relative Luminescence Units (RLU), as described under Materials and methods. IgG concentrations were used to calculate the dilution factor and adjust the amount of the corresponding flow-through (FT, hatched bars) from the purification columns. Results are shown as means ± SEM of 6 measurements from two independent experiments. *P < 0.05 (by the unpaired Student’s *t* test).

We have previously demonstrated that mouse anti-Spike serum could trigger infection of Raji cells [16]. In the context of ADE, antibodies against viral proteins are considered as the major players of enhancement [18,19]; see for review [20,21]. To eliminate the possibility of the participation of other soluble factors during ADE of SARS, in this section we investigated the capability of anti-Spike antibodies alone in enhancing infection of immune cells. We purified IgG by protein G-sepharose chromatography from mouse anti-Spike and control serum, and used 2-fold serial dilutions from 10 to 0.125 μg/mL of the purified portion to form immune complex with SARS-CoVpp and then infected Raji cells. Our results show that purified IgG from mouse anti-Spike serum triggered infection in Raji cells, which was more pronounced with increasing immunoglobulin concentrations (Figure 4). The flow-through (FT) from the same serum, which was diluted by the appropriate factor to be comparable to the final IgG concentration used, elicited no detectable infection at all concentrations (data not shown). Significant differences between ADE of infection with purified mouse anti-Spike IgG and flow-through were observed at concentrations 10 and 2.5 μg/mL, and marginal difference was observed at 0.625 μg/mL. As expected, neither purified IgG (Figure 4) nor flow-through from mouse control serum triggered significant ADE of infection at all concentrations.

### Molecular determinants of FcγRII underlying ADE of SARS-CoVpp

**Figure 5:**
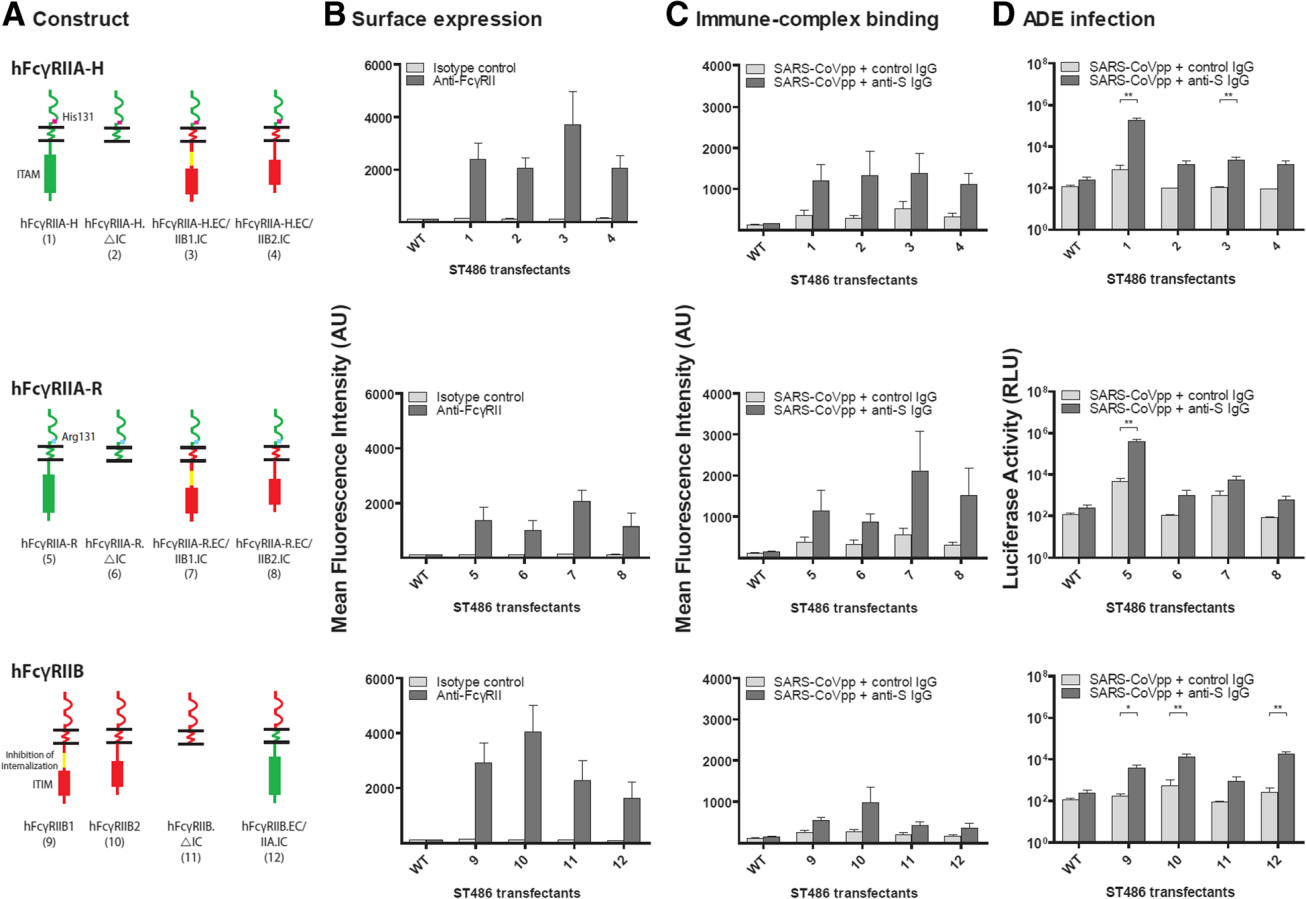
**Susceptibility to ADE is dependent on an intact cytosolic domain. (A)** Schematic representation of wild-type and mutated constructs that were utilized to produce stably transduced ST486 cell lines (see Materials and methods); from top to bottom: human FcγRIIA-His131 (hFcγRIIA-H), FcγRIIA-Arg131 (hFcγRIIA-R), FcγRIIB1 (hFcγRIIB1) and FcγRIIB2 (hFcγRIIB2). **B)** Surface expression in stably transduced ST486 cell lines was evaluated by flow cytometry using a mouse monoclonal anti-FcγRII antibodies (dark grey) or isotype control (light grey), and expressed as mean fluorescence intensity in Arbitrary Units (AU) as described under Materials and methods. Constructs are identified by Arabic numbers and grouped from top to bottom as indicated in **A**. **C)** Immune complex-binding ability of wild-type, truncated and chimeric FcγR receptors. SARS-CoVpp were incubated with purified mouse anti-Spike IgG (dark grey) or control IgG (light grey) to form immune complexes, which were then added to the ST486 transfectants (see Materials and methods). After washing and fixation, bound immune complexes were detected by flow cytometry with an FITC-conjugated goat anti-mouse F(ab’)2, and results shown as in **B**. Constructs are identified by Arabic numbers and grouped from top to bottom as indicated in **A**. **D)** Susceptibility of ST486 transfectants to ADE of infection by SARS-CoVpp. SARS-CoVpp were incubated with purified mouse anti-Spike IgG (dark grey) or control IgG (light grey) and then used to infect cells (see Materials and methods). Cells were washed and three days post infection incubation was stopped by adding luciferase substrate to measure enzymatic activity, which was expressed as Relative Luminescence Units (RLU). Constructs are identified by Arabic numbers and grouped from top to bottom as indicated in A. Results are shown as the means ± SEM of three **(B and C)** or 4–5 independent experiments **(D)**. *P < 0.05; **P < 0.01 by the unpaired Student’s *t* test.

Our recent work had revealed the predominant role of human FcγRII (CD32) in mediating ADE of SARS-CoV [16]. To gain further insight into the underlying molecular mechanism we have investigated the involvement of different domains of FcγRII in mediating ADE. To this end we produced a series of truncated constructs that only carried ectodomain and transmembrane domains of FcγRII, and chimeric constructs with the ectodomain of one receptor and transmembrane and endodomain of another (Figure 5A). We then verified the expression of these constructs on ST486 cells by flow cytometry using a specific monoclonal antibody (clone FLI8.26) that binds to the second Ig-like domain D2γ of FcγRII. Of note, the Fc binding portion of the FcγRII group is very similar among FcγRIIA-H131, FcγRIIA-R131, FcγRIIB1 [22] and also FcγRIIB2, as it harbors identical extracellular structures as FcγRIIB1. Our findings indicate that all FcγRII constructs exhibited detectable expression of FcγRII (dark grey) in ST486 cells in comparison to isotype control (light grey) (Figure 5B). We then tested the ability of the various constructs to bind purified anti-Spike IgG-SARS-CoVpp immune complexes and observed that all ST486 transfectants were able to bind immune complexes (Figure 5C). Finally we investigated whether any difference in susceptibility to SARS-CoVpp ADE of infection was conferred by different FcγRII constructs. When immune complexes formed by 5 μg/mL of purified mouse anti-Spike IgG (the concentration at which the highest infection level was observed in Raji cells) and SARS-CoVpp were added to FcγRII-expressing ST486 cells, all four transfectants expressing wild-type FcγRII forms (cf. FcγRIIA-H vs. FcγRIIA-R, and FcγRIIB1 vs. FcγRIIB2, corresponding to constructs 1, 5, 9, 10, respectively) were infected (Figure 5D), with FcγRIIA-expressing ST486 being more prone to infection than FcγRIIB (cf, constructs 1 and 5 with 9). All the endodomain-truncated constructs (FcγRIIA-H.∆IC, FcγRIIA-R.∆IC and FcγRIIB.∆IC, corresponding to constructs 2, 6, 11 respectively) were not susceptible to ADE of infection, indicating that binding of anti-Spike IgG-SARS-CoVpp immune complexes was not sufficient to mediate entry and that the signaling-competent endodomain was required. However, not all chimeric constructs were able to sustain ADE of infection, suggesting that domain swapping may have partially interfered with signal transduction. Thus only chimeras with FcγRIIA-H ectodomain and FcγRIIB1 endomain, or with FcγRIIB ectodomain and FcγRIIA endomain, exhibited a statistically significant ADE of infection (Figure 5D). This cannot be explained by differences in surface expression or binding of opsonized pseudoparticles, as FcγRIIB.EC/IIA.IC (viz., construct 12) showed robust ADE despite one of the lowest binding ability of immune-complexes.

## Discussion

The possibility that immune response to pathogens may have also deleterious effects on the host homeostasis has been the focus of several studies. For example, the hyper-induction of cytokines following avian influenza infection has been implicated in the severity of the disease [23] and infection of cells by ADE has been known to occur for several viral diseases [20,21]. Here we demonstrate that anti-Spike antibody potentiates infection of human primary immune cells by SARS-CoVpp and replication-competent SARS-coronavirus.

Although we unambiguously obtained evidence of ongoing infection (e.g., *de novo* synthesis of the structural viral protein N), ADE-infected macrophages did not support productive replication of SARS-CoV and, after initiation of viral gene transcription and viral protein synthesis, the replication process stalled ultimately ending in an abortive viral cycle without detectable release of progeny virus. Abortive replication of SARS-CoV into macrophages has already been documented [24] but, at variance with this previous report in which 90% of the macrophages were infected by SARS-CoV in the absence of immune-serum (MOI = 1–2), we observed a much lower infection rate (about 5-7%). One possibility is that such discrepancy may be due to difference of time of sampling (6 hours in our study versus 15 hours in Cheung’s study) and the protocol used for in vitro differentiation (viz., macrophages were differentiated in the presence of fetal calf serum in our study, and autologous plasma was removed two days prior to infection in ref. [8]), leading to difference in infectivity of the cells observed in the studies. Alternatively, we should also consider that the readout of the pseudo-particle experiments was the expression of the luciferase reporter gene, which is under the control of the HIV backbone. This may lead to higher level of protein expression when compared to the abortive replication that follows SARS-CoV infection of macrophages. Thus, the difference may be, in part due to the inability to detect low amounts of Spike protein by immunofluorescence and the difference in sensitivity of the two methods. Of note, the anti-Spike mediated entry is specific for Spike-pseudotyped particles, as shown in Figure 1 of [16].

Because clinical observations have reported poor disease outcomes in early seroconverted SARS patients [25-27], it would be of interest to test SARS patient sera collected at different time-points after SARS onset. However, we have been unable thus far to conduct conclusive assays on a well characterized serum library; moreover, we have to be cognizant of the possibility that ADE may only occur within a narrow window during the course of an infection and only in a subset of infected patients. The alternative possibility that internalization by macrophages may in fact represent an additional mechanism to control viral spread requires further investigation.

In general, studies aiming at better understanding antibody-dependent enhancement of viral infections are focusing on either identifying the (immune) receptor(s) and/or serum component(s) allowing penetration of the pathogen into the target cell, also known as extrinsic ADE, or the outcome response(s) of the target cell downstream to ADE of infection – or intrinsic ADE [20,21,28]. Our previous results demonstrated that only FcγRIIA and, to a lesser extent, FcγRIIB1 triggered infection by SARS-CoVpp in presence of anti-Spike serum [16]. Although it would have been desirable to perform FcγR blocking experiments also in macrophages, co-expression of all FcγRs in these cells [29,30] would cause blocking antibodies to bind not only to the targeted FcγR via their Fab portions, but also to other FcγR via their Fc portion making both FcγRs unavailable for interaction with opsonized pseudoparticles and, consequently, would prevent unambiguous interpretation of the results. We therefore investigated the molecular/signaling requirement underlying FcγRII-mediated infection by SARS-CoV and assessed the relative contribution of the extracellular versus the transmembrane and intracellular domains of the Fcγ receptor II family members in mediating ADE of infection. The extracellular IgG-binding domains of human FcγRIIA and FcγRIIB are closely related, with 78% of homology at the amino acid level. Nonetheless, these 2 receptors have distinct abilities and affinities for the binding of immunoglobulins [31]. Moreover, FcγRIIA carries an immunoreceptor tyrosine-based activation motif (ITAM) whereas FcγRIIB carries an immunoreceptor tyrosine-based inhibitory motif (ITIM), which confer IgG receptors different effector properties [29,30,32]. Altogether, our results demonstrate that binding of SARS-CoVpp to cells via the extracellular domain of FcγRs is not sufficient to trigger ADE of infection as entry requires an intact intracellular domain. Indeed, in spite of roughly similar expression level at the cell surface compared to wild type FcγRII, all mutated FcγRII receptors having truncation of their intracellular domain (FcγRIIΔIC) became unable to trigger ADE of infection while retaining significant level of ligand-bind ability. The results obtained with chimeric constructions where the whole intracellular domain of FcγRIIA and FcγRIIB were swapped lend further support to this interpretation. Of note, the transmembrane and intracellular domains appeared to impart susceptibility to infection of their parental FcγRII. Thus, wild-type FcγRIIA elicited greater ADE than FcγRIIB and grafting the cytoplasmic portion of FcγRIIB reduced susceptibility to ADE of the chimeras with an extracellular FcγRIIA domain to levels comparable to those observed for the FcγRIIA truncated mutants.

However, the relationship between internalization of immune complexes and ADE of infection by SARS-CoV via FcγRIIs appears to be a complex process. Thus, FcγRIIB2 has been shown to mediate internalization of immune complexes at a faster rate than FcγRIIA [33], whereas we found that ADE of infection via FcγRIIA was more prominent than with FcγRIIB. Recently, involvement of downstream signaling triggered by FcγRs activation has been evaluated with respect to ADE of dengue virus infection [34]. Thus, abrogation of FcγRI and FcγRII signaling competency was associated with significant impairment of phagocytosis, but only the signaling-incompetent FcγRI become unable to trigger ADE of dengue virus. Conversely, no discernible effect on dengue virus immune complex infectivity was observed for both wild type and signaling incompetent FcγRIIA. These findings point to fundamental differences between FcγRIA and FcγRIIA with respect to their immune-enhancing capabilities and suggest that different mechanisms of dengue virus immune complex internalization may operate between these FcγRs [34].

## Conclusions

Altogether our results demonstrate that, in presence of vaccine-elicited antiviral antibodies, SARS-CoV displays an altered tropism toward primary human immune cells, which do not express the conventional virus receptor and are otherwise refractory to the virus. A number of SARS vaccine candidates have been tested in experimental animal models [35,36], many of them based on the viral Spike glycoprotein previously identified as the most immunogenic antigen inducing neutralizing and protective antibodies [14,15,37,38]. Of note, some vaccines against animal coronaviruses have been also generated, but their development has proven difficult due to immune enhancement of disease in vaccinated recipients [39-41]. Despite the fact that this alternative infection pathway appears to have a limited impact, it remains of interest to appreciate the cost of antibody-mediated SARS-CoV infection on the functionality of the target cells, in order to have a broad understanding of the tropism and pathogenesis of the virus and evaluate potential pitfalls associated with immunization against human coronaviruses. This aspect is gaining more relevance as the emergence of the MERS-CoV further indicates that the availability of vaccines targeting this group of viruses, which have demonstrated the ability to jump species, is one of the few options available to prevent the spread of infections causing severe diseases with high mortality in humans [42].

## Materials and methods

### Cell lines and expression vectors

The following cell lines used in the study were obtained from the American Type Culture Collection (ATCC; Manassas, VA, USA): HEK293T (human kidney epithelial cells; CRL-11268), Raji (Burkitt’s lymphoma/B lymphoblast), ST486 (Burkitt’s lymphoma/B lymphoblast lacking expression of FcγR; CRL-1647). HEK293T cells were cultured in DMEM supplemented with 10% heat-inactivated fetal bovine serum (FBS), 0.6 mg/l penicillin and 60 mg/l streptomycin, whereas hematopoietic cells were grown in RPMI-1640 supplemented with 10% heat-inactivated FBS, 1% non-essential amino-acids, 4 mM L-glutamine, 1 mM sodium pyruvate, 0.6 mg/l penicillin, 60 mg/l streptomycin, and 20 μM 2-ß-mercaptoethanol (all from Invitrogen Life Technologies, Carlsbad, CA, USA). All cells were maintained at 37°C in a humidified atmosphere with 5% CO_2_ supply.

### Culture of human monocyte-derived macrophages

The research protocol was approved by the Institutional Review Board of the University of Hong Kong/Hospital Authority Hong Kong West Cluster (UW09-375). Blood samples of healthy donors were obtained from the Hong Kong Red Cross Blood Transfusion Service. Human blood cells were isolated from buffy coats and monocyte-derived macrophages were generated in vitro using a modified protocol as previously described [24,43]. Briefly, mononuclear cells were isolated by a Ficoll-Paque density gradient (Pharmacia Biotech, Uppsala, Sweden) to remove erythrocytes, granulocytes, and cell debris. Monocytes were enriched by plastic adherence, harvested, seeded (10^6^ cells/ml) on tissue culture plates and allowed to differentiate for 14 days in the presence of 5% autologous human plasma and 1% fetal calf serum before use. The purity of macrophages was consistently above 90%, as ascertained by immunofluorescence staining for human CD68, a lysosomal glycoprotein that is highly expressed by macrophages and monocytes [44,45].

### Production of anti-Spike immune serum

BALB/c mice were immunized with recombinant SARS-CoV Spike proteins adjuvanted with alum, as previously described [15,16]. Saline-injected mice served as controls. Serum was collected at day 55 post-immunization, heat-inactivated for 30 min at 56°C and stored at −20°C for subsequent use.

### Production and use of lentiviral particles pseudotyped with SARS-CoV Spike

The protocol to produce SARS-CoV pseudotyped lentiviral particles expressing a luciferase reporter gene (SARS-CoVpp) has been described elsewhere [17]. Following a purification step on 20% sucrose cushion, the concentrated viral particles were titrated by ELISA for lentivirus-associated HIV-1 p24 protein according to the manufacturer’s instruction (Cell Biolabs, Inc., San Diego, CA, USA), and stored at −80°C until use. For ADE assays, heat-inactivated mouse anti-Spike or control serum was incubated for 1 hr at 37°C with SARS-CoVpp. This inoculum was deposited on cells and infection proceeded for 1 hr at 37°C. Following repeated washing cells were incubated for additional 60–65 hours and then fixed in 4% paraformaldehyde (Sigma-Aldrich Inc., St. Louis, MO, USA) for immunofluorescence microscopy.

### Infection with replication-competent SARS-CoV

Laboratory procedures involving replication-competent viruses were performed in biosafety level-3 containment (State Key Laboratory of Emerging Infectious Diseases, The University of Hong Kong). The SARS-CoV strain used (HK39849) is a clinical isolate [46], which was cultured in fetal rhesus kidney-4 (FRhK-4, ATCC code CRL-1688) cells. In ADE assays, heat-inactivated mouse anti-Spike or control serum was incubated for 1 h at 37°C with SARS-CoV and human monocyte-derived macrophages were infected at an MOI of 1 for 60 min at 37°C. After repeated washings, cells were cultured with fresh medium (time 0) supplemented with 2% FBS for the indicated time points (hours) post infection (p.i.) and eventually either fixed in 4% paraformaldehyde (Sigma-Aldrich Inc.) for immunofluorescence microscopy, or resuspended in lysis buffer (RLT buffer, RNeasy RNA Mini kit; QIAGEN, Germantown, MD, USA) for real-time quantitative RT-PCR. Samples of cell culture supernatants harvested at different h.p.i. were also mixed with RLT buffer and processed as above for RT-PCR.

### Immunofluorescence microscopy

Infectivity of replication-competent SARS-CoV was determined by indirect immunofluorescence with a mouse monoclonal antibody directed against SARS-CoV nucleocapsid protein (clone 4D11; a gift from Dr. Kwok-Hung Chan, Department of Clinical Microbiology, Queen Mary Hospital, Hong Kong), at a dilution of 1:200. Staining was revealed with a tetramethylrhodamine-5-(and-6)-isothiocyanate (TRITC) conjugated goat anti-mouse antibody (Invitrogen Life Technologies) on an AxioObserver Z1 microscope (Carl Zeiss, Inc., Thornwood, New York, USA). Pictures taken from 5 randomly chosen fields at a magnification of 400X were acquired with an Axiocam MRm camera and analyzed with Metamorph software (Molecular Devices, Sunnyvale, CA, USA). Infectivity was determined as the percentage of cells expressing viral antigen. Similar staining procedures were employed for assessing infection by SARS-CoVpp, except for the use of direct immunofluorescence with a fluorescein isothiocyanate (FITC) conjugated goat monoclonal antibody specific for firefly luciferase (Rockland, Gilbertsville, PA, USA) at a dilution of 1:100.

### Real-time quantitative RT-PCR for viral gene expression

RNA was extracted with the RNeasy RNA Mini kit (QIAGEN), according to the manufacturer’s recommendations; concentration and purity of RNA were measured by standard optical methods. Reverse transcription was performed on total RNAs with Superscript III reverse transcriptase as specified by the manufacturer (Invitrogen Life Technologies). For quantification of SARS-CoV gene copies, cDNA was generated in separate reactions in presence of either forward or reverse primers specific for the nucleocapsid and ORF1b genes (Table 1) that amplified negative and positive strand viral RNAs, respectively. This assay allows distinguishing between signals generated by entry of input virus from downstream events reflecting an active replication process [47]. Specific primers (Table 1) and TaqMan Minor Groove Binder probes used for detection of SARS-CoV nucleocapsid gene have been previously described [48]. However, it should be noted that the primer set used for the nucleocapsid gene could also detect the negative strand of the viral genomic RNA and could not distinguish it from sub-genomic material. The qPCR assay was carried out in a final volume of 20 μl and the fluorescence signal was detected with a LightCycler 480-II (Roche Applied Science, Mannheim, Germany) programmed as follows: 95°C for 10 min, followed by 45 cycles of 95°C for 10 sec, 60°C for 30 sec, and 72°C for 1 sec. Results were expressed as the number of target copies per 10^8^ copies of the 18S rRNA gene, which was used to normalize results. To ensure the consistency of qPCR measurements over the time period of the study, the same batches of primers and probes were employed. The qPCR results were considered valid when the efficiency of the standard curve was between 1.9 to 2.1 and the R^2^ value was greater than 0.99.

**Table 1:** Sequences of primers and probes used in real-time qPCR assay and cDNA synthesis

**Gene**	**Sequences**	**Amplification product (bp)**	**GeneBank accession number**
18S rRNA	(F) 5′-CggAggTTCgAAgACgATCA	90	NR_003286
	(R) 5′-ggCgggTCATgggAATAAC		
	(P) 5′(HEX) ATACCgTCgTAgTTCCgACCA (BHQ)		
Nucleocapsid	(F) 5′-ACCAgAATggAggACgCAATg	85	AY278491
	(R) 5′-gCTgTgAACCAAgACgCAgTATTAT		
	(P) 5′(FAM) ACCCCAAggTTTACCC (NFQ)		
ORF1b	(F) 5′-CAgAACgCTgTAgCTTCAAAAATCT	68	AY278491
	(R) 5′-TCAgAACCCTgTgATgAATCAACAg		
	(P) 5′(FAM) TCTgCGTAggCAATCC (NFQ)		

F: forward primer; R: reverse primer; P: TaqMan probes; 18S rRNA: 18S ribosomal RNA.

### Purification of IgGs from mouse serum

IgGs was purified from immunized and control mice using protein G-sepharose (GE Healthcare, Little Chalfont, United Kingdom) according to the manufacturer’s recommendations. The purity of the IgG fraction was checked by silver staining following gel electrophoresis and the concentration of anti-Spike IgG determined by ELISA.

### Construction of lentiviral vectors for wildtype, endodomain-truncated and chimeric forms of FcγRII (hCD32)

The strategy to produce plasmids for wild-type human IgG receptors (FcγRs) consisted in replacing the enhanced green fluorescent protein (eGFP) gene from the bicistronic vector pCHMWS-eGFP_IRES_Hygromycin (a kind gift from Drs. Rik Gijsbers and Zeger Debyser, Katholieke Universiteit Leuven, Belgium) with the coding sequences for FcγRIIA (hCD32a) isoforms (FcγRIIA.R131 and FgRIIA.H131 GenBank accession No. NM_021642) and FcγRIIB1 (hCD32b; GenBank accession No. AF543826) flanked by BglII and SalI sites, as described elsewhere [16]. The synthetic sequences (GeneArt, Regensburg, Germany) were inserted into the original transfer plasmid to yield wild-type constructs. pCHMWS-FcγRIIB2_IRES_Hygromycin was constructed by swapping the intracellular domain of FcγRIIB2, obtained from RT-PCR amplification of the desired sequence from polyclonal Raji cDNA, with the corresponding region of the pCHMWS-FcγRIIB1_IRES_Hygromycin construct. Wild-type constructs were subsequently used to generate truncated and chimeric forms of FcγRIIs by standard techniques (see Figure 5A for a schematic representation of the mutants tested). All plasmids were sequenced by the Centre for Genomic Sciences of the University of Hong Kong.

### Generation of stable ST486 cell lines expressing wild-type and mutated FcγRs using lentiviral particle-based gene transduction

Cell lines were generated by transduction of monoclonal ST486 cells with vesicular stomatitis virus (VSV) G pseudotyped lentiviral particles bearing the specified transgene as previously described [16]. At 2 days post-infection, cell surface expression of the FcγRs was monitored by flow cytometry and cells were subsequently cultured in selective medium containing 250 μg/ml hygromycin (Invitrogen Life Technologies) when appropriate. Finally, several monoclonal cell lines for each construct were isolated and expression of the transgene was confirmed by flow cytometry.

### Evaluation of FcγR expression on ST486 transfected cell lines by flow cytometry

Expression of FcγRIIs was evaluated by flow cytometry as described in [16]. The following mouse MAbs were used: 3G8 anti-hCD16, FLI8.26 for hCD32, and 10.1 anti-hCD64 (all from BD PharMingen, Frankin Lakes, NJ, USA); MOPC-21 (IgG1, κ) or MPC-11 (IgG2b, κ) isotype controls (both from BioLegend, San Diego, CA, USA). Cells were washed and primary antibody binding was revealed by staining on ice for 30 min with FITC-conjugated goat anti-mouse antibodies (Jackson ImmunoResearch, West Grove, PA, USA). Data were collected from at least 10,000 singlet living cells on a LSRII flow cytometer (BD Biosciences, San Jose, CA, USA) and analyzed using FlowJo software (TreeStar, Ashland, OR, USA).

### Binding of immune complexes to FcγRII

SARS-CoVpp-IgG immune complexes were obtained by incubating SARS-CoVpp with 30 μg/ml of either purified mouse anti-Spike or control IgG at 37°C for 1 hr. The mixture was then quickly chilled on ice for 10 min and added (100 μl/well) to ST486 cells (3×10^5^ cells/well), which had been previously stained with 0.1% fixable viability dye eFluor 660 (eBioscience, San Diego, CA, USA). Following a 1 hr incubation on ice, cells were washed twice with cold PBS, fixed with 1% paraformaldehyde for 20 min on ice and immune complex binding was revealed by staining with 5 μg/ml FITC-conjugated goat anti-mouse F(ab’)_2_ (Jackson ImmunoResearch) at 4°C for 30 min. Data were collected and analysed as described above.

### Statistical analysis

Results are shown as means ± SEM of the indicated number of observations. Statistical difference between groups was determined by the unpaired Students’s *t*-test with a 0.05 significance level.

## Abbreviations

ACE2: Angiotensin-converting enzyme-2; ADE: Antibody-dependent enhancement; DC-SIGN: Dendritic cell specific intercellular adhesion molecule 3-grabbing non-integrin; eGFP: Enhanced green fluorescent protein; FITC: Fluorescein isothiocyanate; h.p.i.: Hours post infection; L-SIGN: Liver/lymph node-specific intercellular adhesion molecule-3-grabbing integrin; ITAM: Immunoreceptor tyrosine-based activation motif; ITIM: Immunoreceptor tyrosine-based inhibitory motif; MERS-CoV: Middle East respiratory syndrome coronavirus; MOI: Multiplicity of infection; RT-PCR: Reverse transcriptase-polymerase chain reaction; SARS-CoV: Severe acute respiratory syndrome coronavirus; SARS-CoVpp: Evere acute respiratory syndrome coronavirus pseudo-particles; TRITC: Tetramethylrhodamine-5-(and-6)-isothiocyanate; VSV: Vesicular stomatitis virus.

## Competing interests

The authors declare that they have no competing interests.

## Authors’ contributions

MSY, NHLL, PHL, HHYL performed cell culture, molecular biology, microscopy, and flow cytometry studies; CYC performed the experiments with SARS-CoV in the BSL-3 lab; MJ, MSY, NHLL, MD, JSMP, RB participated in the design of the study and analysis of the data; MJ and RB wrote the manuscript. All authors commented and approved the final version of the manuscript.
